# Segmented-Block Poly(ether amide)s Containing Flexible Polydisperse Polyethyleneoxide Sequences and Rigid Aromatic Amide Moieties

**DOI:** 10.3390/ma14112804

**Published:** 2021-05-25

**Authors:** Miriam Trigo-López, José A. Reglero Ruiz, Patricia D. Peredo, Aranzazu Mendía, Asunción Muñoz, Félix C. García, José M. García

**Affiliations:** Departamento de Química, Facultad de Ciencias, Universidad de Burgos, Plaza de Misael Bañuelos s/n, 09001 Burgos, Spain; jareglero@ubu.es (J.A.R.R.); pdperedo@ubu.es (P.D.P.); amendia@ubu.es (A.M.); amugnoz@ubu.es (A.M.); fegarcia@ubu.es (F.C.G.)

**Keywords:** condensation monomers, aromatic polyamides, polyethyleneoxide, segmented block copolymers

## Abstract

We describe the synthesis and characterization of three novel aromatic diamines containing oxyethylene sequences of different lengths. These diamines were polymerized using the low-temperature solution polycondensation method with isophthaloyl chloride (IPC), terepthaloyl chloride (TPC), [1,1’-biphenyl]-4,4’-dicarbonyl dichloride (BDC), and 4,4′-oxybis(benzoyl chloride) (OBE), obtaining twelve poly(ether amide)s with short segments of polydisperse polyethyleneoxide (PEO) sequences in the polymer backbone. These polymers show reasonably high molecular mass materials (Mw > 12,000), and the relationship between their structure and properties has been carefully studied. Compared with conventional polyamides containing monodisperse PEO sequences, the polydispersity of the PEO segments within the structural units exerts a significant influence on the crystallinity, flexibility, solubility, and the thermal properties of the polymers. For instance, the all-*para* oriented polyamides (TPCP-A), with an average number of 8.2 ethylenoxide units per structural unit can be transformed conventionally (T_m_ = 259 °C) in comparison with thermally untransformable polymer with 2 ethylenoxide units (T_m_ = 425 °C).

## 1. Introduction

Aromatic polyamides are characterized by their excellent balance of thermal and mechanical properties. They are valuable materials for engineering applications (mainly as high-strength fibers, films, or coatings for various fields, including the automotive and aerospace industries, bullet-proof protection, electronics, etc.). However, they are challenging to process because they do not melt (or their melting temperatures are out of the conventional thermal transformation techniques or in the limit of degradability) and do not dissolve in common organic solvents, limiting their use. Therefore, many efforts have been made to chemically modify the structure of aromatic polyamides to increase their solubility in organic solvents or/and lower their transition temperatures to improve their processability to broaden the scope of their applications [1,2]. Some of these efforts include the introduction of bulky side groups [3,4,5], the introduction of asymmetrically substituted meta-oriented rings [6,7], or flexible chains in the polymer backbone such as ether linkages [8,9,10].

In a step forward, it is possible to prepare poly(ether-block-amide)s to be used in many applications [11], such as membranes for gas separation [12,13,14,15], antistatic agents [16], or intelligent packaging [17]. Some examples include the synthesis of different segmented block poly(ether amide) copolymers containing aromatic polyether blocks [18] or polyethyleneoxide sequences with a fixed number of ethyleneoxide units in each polymer [19,20]. Regarding the latter, different families of polyamides containing short segments of polyethyleneoxide, from two to six units, alternating with short and rigid moieties of aromatic polyamides were previously studied. These polymers showed unexpected high melt transition temperatures and high crystallinity. It was ascribed to the high mobility of the ether linkage of the glycol moieties, the amide groups’ hydrogen bonds, and the molecular regularity provided by the fixed length of the oxyethylene spacers in each polymer. Thus, the increase in the number of oxyethylene units from two to six was neither enough to lower the melt transition temperatures significantly nor to improve the solubility compared to commercial aramids, mainly because of the high tendency of the polymers to crystallize [20].

Our goal is to prepare poly(ether amides) with aromatic diamine monomers to keep thermal and mechanical stability but, this time, using polydisperse poly(ethyleneoxide) to avoid the regularity of the fixed-length oxyethyele spacers. Therefore, this work deals with the synthesis of a new family of segmented block poly(ether amide)s containing short sequences of polydisperse poly(ethyleneoxide), alternating with short moieties of aromatic polyamide. The main objective of this study was the search for aliphatic–aromatic poly(ether amide)s that could offer a balance of properties between those of aliphatic nylons and aromatic polyamides, which means the obtention of materials with improved transformability without impairing the critical thermal and mechanical performance. A significant innovative aspect has been synthesizing three polydisperse diamines containing short sequences of polydisperse polyethyleneoxide with *meta* or *para* diaminophenoxy end groups. The diamines were reacted with several diacid chlorides to render poly(ether amide)s with a random distribution of oxyethylene sequences of different lengths. The random distribution of oxyethylene segments of different lengths between amide moieties was expected to promote lower crystallinity, higher flexibility without using plasticizers, lower transition temperatures, and better solubility of the resulting polymers, improving their transformability and expanding their applications. As a result, these new polymers might be an alternative for aliphatic poly(ether amide)s, currently used as 3D printable materials, in medical and sports equipment, electronic goods, etc. [21,22], through the combination of flexibility and low density with good thermal and mechanical stability due to the presence of aromatic rings. Furthermore, through a good mix of the aramid and the polyether segments, various end-use performance characteristics are possible.

## 2. Materials and Methods

### 2.1. Materials

All materials and solvents were commercially available, and they were used as received unless otherwise indicated. Terephthaloyl chloride (TPC) (≥99%, Sigma-Aldrich, St. Louis, MO, USA) was recrystallized twice from dry heptane (99.9%, VWR-Prolabo, Fonteney-sous-bois, France). *N,N*-Dimethylacetamide (DMA) (99%, Sigma-Aldrich was vacuum distilled twice, once over phosphorous pentoxide and the second time over calcium hydride. It was stored in a sealed, dark flask over molecular sieves.

[1,1’-biphenyl]-4,4’-dicarbonyl dichloride and 4,4′-oxybis(benzoyl chloride) were synthesized and purified as described elsewhere [23]. Polyethyleneoxide 400 was supplied by Aldrich and was characterized by gas chromatography–mass spectrometry methods. The average molecular mass was 357 Da (M_n_), which corresponds to an average sequence of eight oxyethylene units.

### 2.2. Methods

^1^H and ^13^C NMR spectra were recorded on a VARIAN UNITY INOVA 400 (Varian, Palo Alto, CA, USA), operating at 399.94 MHz in ^1^H and 10.55 MHz in ^13^C, with DMSO-*d*_6_ as the solvent, and tetramethylsilane (TMS) as an internal reference.

Differential scanning calorimetry (DSC) analyses were performed on a Perkin-Elmer DSC calorimeter (PerkinElmer, Inc., Waltham, MA, USA) at a heating rate of 20 °C/min under nitrogen. In general, two runs were needed for achieving fully comparable results.

Thermogravimetric analyses (TGA) were performed on a Perkin-Elmer TGA thermobalance (PerkinElmer, Inc., Waltham, MA, USA). Measurements were performed on 2 ± 0.5 mg samples heated in flowing nitrogen at 10 °C/min. The samples were previously dried under vacuum at room temperature for 24 h over phosphorous pentoxide.

Inherent viscosities were determined at 25.0 ± 0.1 °C with a manual Ubbelohde viscometer using TFA/CHCl_3_ (1/1) (SI Analytics, Hattenbergstr, Germany, country) as a solvent for all polymers. The polymer concentration was 0.5 g/dL.

The polymer solubility was evaluated at 20 °C with 10 mg of the polymer and 1 mL of the solvent and stirring for 24 h. The polymer is considered soluble at room temperature if a homogeneous solution is observed; otherwise, the system is heated to reflux for two hours, considering the polymer soluble on heating if a homogenous solution is formed. The polymer is deemed to be insoluble if no homogeneous solution is observed after that.

The solution–evaporation casting procedure prepared polymer films in NMP as the solvent with about 7% by polymer mass. After the solvent was evaporated in an air-circulating oven, the films were washed with distilled water. The measurements of mechanical properties were performed with these untreated films with a Shimazdu equipment (EZ Test Compact Table-Top Universal Tester, Shimadzu, Kyoto, Japan). The films were cut in 5 × 40 mm^2^ strips. Measurements were performed at a 5 mm/min extension rate and 9.44 mm gauge length, using five samples of each film and then averaging the data.

The RAMAN spectra were taken using a confocal AFM-RAMAN model Alpha300R–Alpha300A AFM from WITec (WITec Wissenschaftliche Instrumente und Technologie GmbH, Ulm, Germany). In the punctual RAMAN experiments, a laser of 532 nm of wavelength was used, using a laser intensity of 5 mW. All spectra were taken at rt and at a magnification of 100X. Infrared spectra (FTIR) were registered with a Jasco Spectrometer FT/IR-4200 FTIR (Jasco, Victoria, Canada) with an ATR-PRO410-S accessory.

For the AFM measurements, areas of 25 and 1 μm^2^ were analyzed. AFM worked in tapping mode, using a driving amplitude of 0.25 V and a tip of 42 N/m. Each area took around 5 min of data acquisition. Surface rugosity mappings, derived from the tip penetration measurements, were extracted using the WITec software. Two different experiments were carried out. First, surface rugosity was analyzed at rt for all the samples. Secondly, a surface mapping was collected after heating the samples at four different temperatures (Each sample was heated in a furnace up to the desired temperature, stabilized during 30 min, and suddenly quenched in a salted ice water bath at −10 °C. Then, after drying at rt, each sample was measured separately in the AFM equipment).

XRD spectra were collected using a Bruker D8 discover equipment (Bruker, Billlerica, MA, USA). Each spectrum was acquired from 5° to 80°, using a step size of 0.05° (2θ). Wavelength of X-ray radiation was 1.54060 nm, with an intensity of 30 mA. The first set of measurements were carried out at rt for all the films, whereas the XRD spectra film IPCP-A was also analyzed at different temperatures to correlate the results with the AFM mapping images. In this case, a single film sample was placed in the chamber and heated up to the desired temperature. After 30 min of stabilization, the XRD spectrum was collected.

Gel permeation chromatography (GPC) analyses were performed using PLgel columns (Agilent, Santa Clara, CA, USA), nominal pore sizes: 500, 1 × 10^4^, and 1 × 10^5^ Å; solvent: *N,N*-dimethylformamide (DMF) with 0.1 % of LiBr; temperature of the columns: 70 °C; flow rate: 1 mL/min; detector: UV). The columns were calibrated with narrow, homemade samples of poly(*m*-phenylene isophthalamide) [19].

### 2.3. Synthesis of Monomers and Polymers

#### 2.3.1. Synthesis of α,ω-Dichloropolyethylenglycol (**1**)

**Scheme 1:**
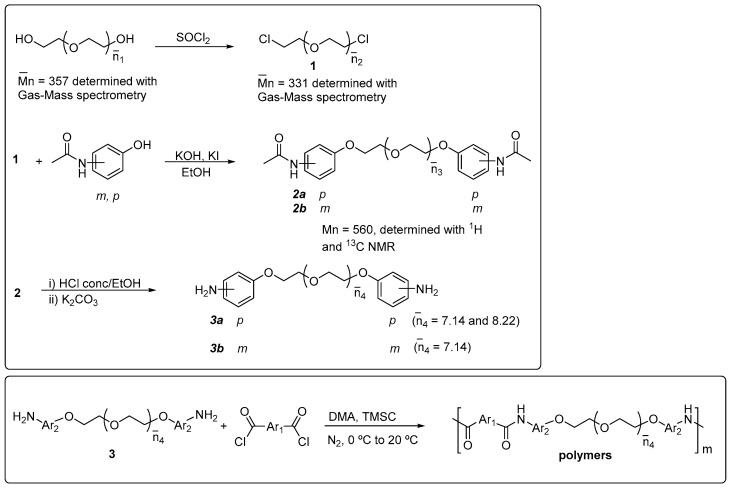
Polymer synthesis, structure, and codes of poly (ether amide)s.

Hydroxy-terminated polyethyleneoxide was converted into the α,ω-dichloro derivative by the following general method: a mixture of 300 mL of dry hexane, 100 g (0.84 mol) of thionyl chloride, and two drops of DMF were cooled to 10 °C in a 1 L round flask fitted with a condenser and a mechanical stirrer. Then, 100 g (0.28 mol) of polyethyleneoxide 400 (M_n_ = 357 Da) was added dropwise for 4 h. Next, the mixture was heated to reflux, and the reaction was allowed to proceed for 2 h more. The thionyl chloride and the hexane were removed under vacuum. The pale-yellow liquid obtained was washed three times with 0.1 N NaOH and several times more with distilled water in a separatory funnel. After that, the product was isolated and dried with anhydrous sodium sulfate. A colorless liquid was obtained, which was used in the next step without further purification. The average number of ethyleneoxide sequences (M_n_ = 331 Da) was measured with a gas chromatography–mass detector. M_n_ of **1** is lower than the M_n_ of the starting hydroxy-terminated polyethyleneoxide a result of the generation of lower-molecular-weight species caused by bond breaking due to the presence of SOCl_2_ and the generation of HCl in the reaction media. In that way, species containing from two to nine ethyleneoxide units could be detected (Scheme 1). Monodisperse standards for calibration were synthesized previously [19].

#### 2.3.2. Synthesis of the Diamines (**3**)

Polydisperse α,ω-bis(4-aminophenoxy)polyethyleneoxide (**3a**). A mixture of 80.1 g (0.53 mol) of 4-acetamidophenol, 200 mL of ethanol, 29.74 g (0.53 mol) of KOH, 0.1 g of KI, and 100 mL of water was heated at 50 °C in a 500 mL round flask fitted with a mechanical stirrer and a condenser. Then, 79.4 g (0.24 mol) of the correspondent polydisperse dichloropolyethyleneoxide **1** (M_n_ = 331 Da) was added dropwise for 5 h, and the reaction was continued for 48 h more. The solvent was removed by vacuum distillation to render a dark-colored solid that was dissolved in 150 mL of dichloromethane. The solution was washed with five portions of 100 mL of water to remove salts and the excess of potassium 4-acetamidophenoxide. The solvent was removed in a rotary evaporator to obtain 94.1 g (70 %) of the polydisperse diacetamido intermediate **2a**.

To 50 g (0.09 mol) of the polydisperse bis-diacetamido product, **2a**, 500 mL of ethanol and 200 mL of conc. HCl were added. Then, the mixture was heated to reflux for 2 h in a 1 L round flask fitted with a condenser and a magnetic stirrer. Upon cooling, the ethanol was distilled. The solution was neutralized with K_2_CO_3_ to obtain a dark, oily product separated from water in a separatory funnel, dissolved in MeOH and treated with charcoal. The charcoal was filtered off, and the methanol was vacuum distilled to render the crude polydisperse diamine **3a**. Yield: 31 g (73 %). The diamine was purified in a silica gel chromatography column (ethylacetate/hexane: 95/5), giving rise to two fractions of aromatic diamines with short segments of polydisperse polyethyleneoxide (PEO) sequences (average number of PEO sequences of 8.22 and 7.14).

Polydisperse α,ω-bis(3-aminophenoxy)polyethyleneoxide (**3b**). We used the same synthetic route given above for bis(4-aminophenoxy)ethylenglycol starting with 3-acetamidophenol. The diamine was purified in a silica gel chromatography column (ethylacetate/hexane: 95/5), giving rise to a fraction of aromatic diamines with short segments of polydisperse polyethyleneoxide (PEO) sequences (average PEO sequences of 8.22).

#### 2.3.3. Synthesis of Polymers

All polymers were synthesized using the same general method as follows: in a 100 mL double-walled glass reactor fitted with a mechanical stirrer, diamine (10 mmol) and trimethylsilyl chloride (TMSC, 10 mmol) were dissolved in 20 mL of DMA at room temperature. The solution was stirred for 0.5 h, and then cooled to 0 °C by a circulating cooling system, while a blanket of dry N_2_ was maintained. Diacid chloride (20 mmol) was added portion-wise to the stirred, cooled solution, and the mixture was allowed to react under nitrogen at 0 °C for 1 h and at 20 °C for 2h. The solution was cooled to 0 °C again, and a small excess (1%) of the diacid chloride was added. Cooling was discontinued, and the reaction was stopped after 2 h at room temperature. The final solution was poured into distilled water, producing a white, fibrous polymer precipitate. The polymer was washed with water and extracted in a Soxhlet with acetone for 24 h and then dried in a vacuum oven at 70 °C overnight. Polymer yields were quantitative. Viscosities are summarized in Table 2.

## 3. Results and Discussion

The preparation of aromatic diamines containing a fixed number of oxyethylene sequences has been previously reported. Thus, various polymers have been synthesized with unique spacer lengths between amide and imide groups [19,20].

In the present study, we have synthesized three diamine monomers, consisting of a mixture of aminophenyl-terminated polyethyleneoxides containing two to nine units of ethyleneoxide. The synthetic route to these monomers is given in Scheme 1, together with the obtained polymers (Table 1). The starting material for these new diamines is a commercial polydisperse polyethyleneoxide with an apparent molecular mass of 400. The average number of ethyleneoxide units was determined in the first synthetic steps by gas–mass chromatography and by ^1^H NMR and quantitative ^13^C NMR spectroscopy in the later synthetic steps.

**Figure 1:**
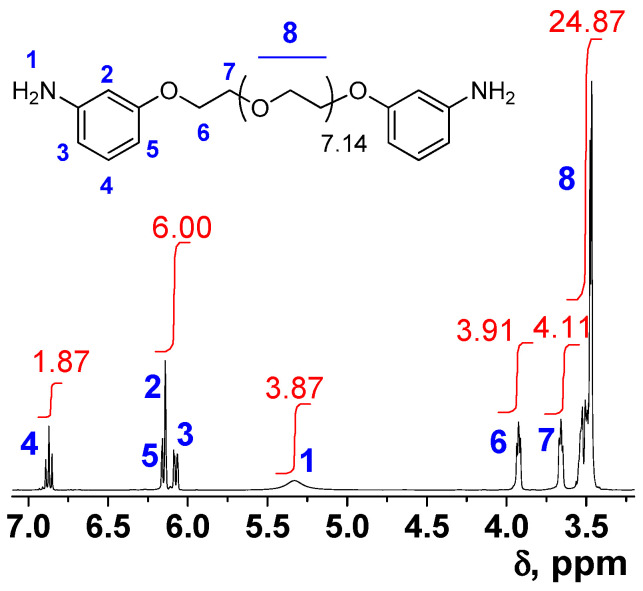
^1^H NMR of *meta*-oriented diamine **3b** (in blue and red numbers the hydrogen assignation and the integrals, respectively).

We aim to study the influence in the polymer properties of the average number of oxyethylene units (*soft* segments) in the polymer backbone while maintaining the rigid amide moieties (*hard* segments). For this purpose, two fractions of pure *para*-oriented diamine **3a** with a different average number of ethyleneoxide sequences were collected from the silica gel purification column. The two diamine fractions have an average number of ethyleneoxide units of 7.14 and 8.22, and an average molecular mass number of 515 and 563, respectively. However, only one fraction of diamine was collected in the purification step of *meta*-oriented diamine **3b** by column chromatography, with an average number of ethyleneoxide units of 8.22 and the average molecular mass 562. As described in Figure 1 for the *meta*-oriented amine, **3b,** where the proton signals were assigned, the relationship between aromatic and aliphatic proton area in ^1^H NMR was employed to estimate the average molecular mass of each fraction.

**Figure 2:**
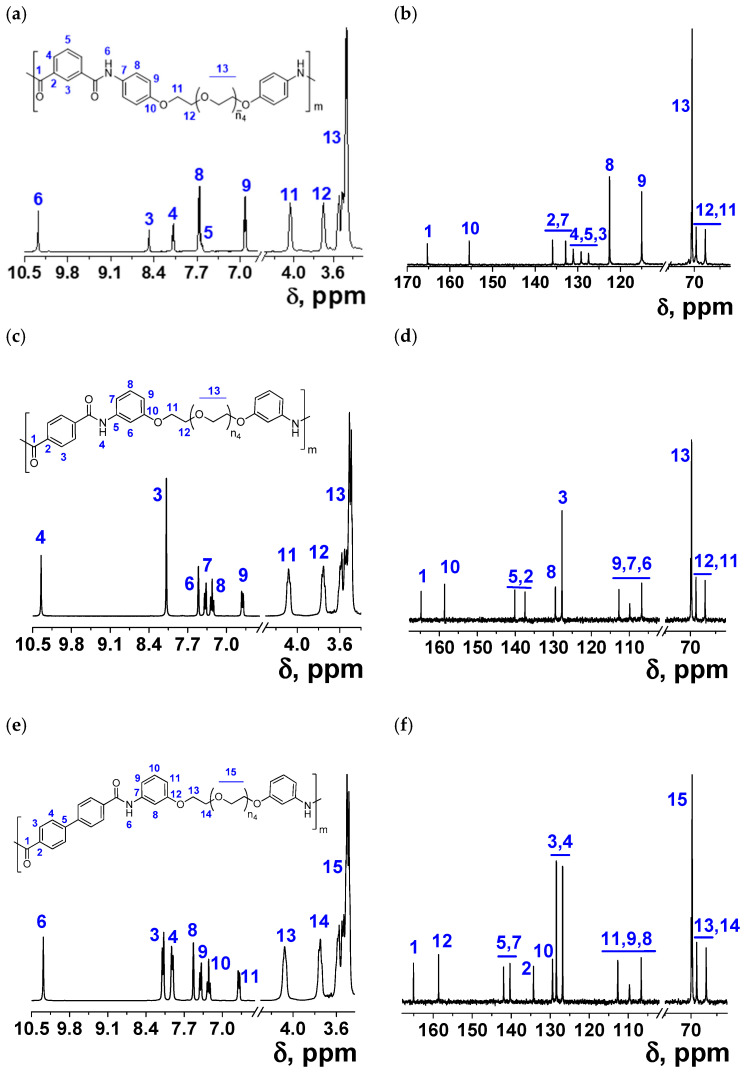
NMR spectra of IPCP-A (n_4_ + 1 = 8.22) (**a**) ^1^H NMR and (**b**) ^13^C NMR; NMR spectra of TPCM (n_4_ + 1 = 8.22) (**c**) ^1^H NMR and (**d**) ^13^C NMR; and NMR spectra of DBCM (n_4_ + 1 = 8.22) (**e**) ^1^H NMR and (**f**) ^13^C NMR.

For the preparation of polyamides, IPC, TPB, BDC, and OBE were reacted with the three diamines to obtain twelve polymers (Scheme 1 and Table 1). The diamines were previously silylated in situ to increase the nucleophilicity of the amine group toward acid chloride. Thus, the polycondensation of monomers was carried out in solution at 0 °C using DMA as a solvent and TMSC as a molecular mass promoter. Results obtained in the synthesis of the polyamides are shown in Scheme 1. The range in the inherent viscosities of all the polymers was between 0.44 and 1.21 dL/g (Table 2). Spectroscopic methods were used to identify monomers and polymers. As examples, the ^1^H NMR and ^13^C NMR spectra of polymers IPCP-A, TPCM, and DBCM have been reproduced in Figure 2, where the proton and carbon signals have been assigned and the structures consequently confirmed.

The solubility of these polyether amides was tested in various solvents (see Table 3), according to the procedure described in Section 2. All polymers were soluble in polar aprotic solvents, such as NMP. The solubilities are compared with those of poly(ether amide)s that do not have a distribution of PEO sequences in the main chain. As expected, these novel polyamides showed improved solubility compared to those with a fixed length between rigid aromatic moieties. This may be attributed to the lower crystallinity and the lower cohesive energy coming from the impairing of intrachain hydrogen bond formation (because of the nonregular distribution of amide groups along the main polymer chain of the polyamides with longer and dispersed PEO sequences).

On the other hand, solubility is strongly dependent on the chain rigidity, and thus, the increase in the number of *para*-substituted rings contributes to a lower solubility level. The number average of oxyethylene units in the diamine moiety is also crucial. In all cases, the lengthening of the polyethyleneoxide sequence produced a higher solubility, as can be observed in the series A and B derived from *para*-substituted diamines.

**Figure 3:**
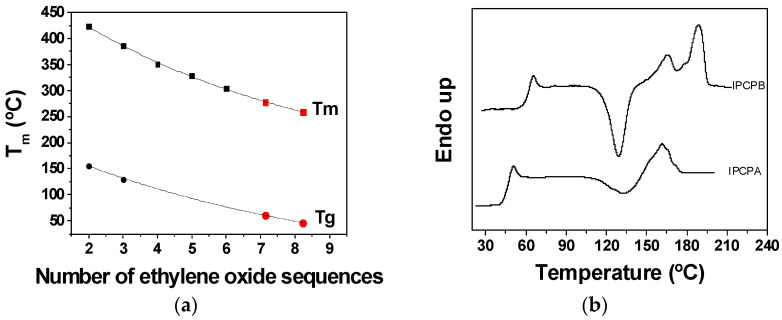
Thermal properties of poly(ether amides) as a function of the ethyleneoxide sequences: (**a**) glass transition temperatures of polymers TPCP-A and TPCP-B related to the number of ethyleneoxide sequences (T_g_ and T_m_ data for n = 2–6 was obtained from the literature, black symbols [20]; data from this work correspond to the number of ethyleneoxide sequences of 7.14 and 8.22, red symbols); (**b**) DSC of polymers IPCP-A and IPCP-B.

The melt transition temperature is also directly dependent on the number of ethyleneoxide units. In Figure 3, the influence of the spacer length on the T_m_ can be seen. The glass transition temperatures were also affected by the length of the *soft* segments. As a rule, T_g_ values were much lower than those of poly(ether amides) containing ethyleneoxide spacers of fixed length (Figure 3a and Table 2) [20]. This is consistent because polydisperse poly(ether amides) could not provide as many possibilities for establishing interchain hydrogen bonds as regular poly(ether amide)s can. These T_m_s, and the T_d_s, allow for the transformation of the polymer by conventional techniques, such as injection molding or extrusion. For instance, the T_m_ of a well-known thermoplastic, such as PET (polyehtylene terephthalate), is about 255 is °C, which can be well compared with the T_m_ of TPCP-A, 259 °C. PET is transformed at a barrel temperature of about 280–300 °C. The influence of the length of ethyleneoxide sequence can be observed in Figure 3b, where DSC of polymers IPCP-A and IPCP-B are depicted as an example. IPCP-A film (Figure 3b) shows three thermal transitions: glass transition temperature around 50 °C, crystallization at 135 °C, and a melting region around 165 °C. Shortening the length of the ethyleneoxide sequences (IPCP-B) results in higher melting transitions. This way, the glass transition temperature of IPCP-B rises to 65 °C, and the second melting transitions between 167 and 180 °C.

The decomposition temperature (T_d_, Table 2) is lower by 20 °C compared to polymers with fixed sequences PEO (six PEO units) [20]. This fact can be attributed again to the diminution of hydrogen bonds per volume unit (hence, lower cohesive energy) and to the relative increase of the aliphatic compared with the aromatic moieties in the polymer chain. All polymers showed a narrow range of T_d_s that did not allow for finding any trend based on the rigid amide moiety molecular structure, which supports that the initial decomposition steps take place in the *soft* PEO segments.

To exemplify the mechanical properties of the new poly(ether amide)s, cast films of IPCP-A, DBMC, and TPCM were prepared. The results described in Table 4 showed Young’s modulus and tensile strength within the same range for the three polymers and are consistent with introducing flexible spacers between the rigid segments. These values are considerably lower than the ones obtained for commercial poly(*m*-phenylene isophthalamide) prepared in the same conditions (Young’s modulus 1.4 GPa and tensile strength 63 MPa) [24], bringing to light the flexibility of the synthesized polymers due to the presence of flexible segments, as expected.

**Figure 4:**
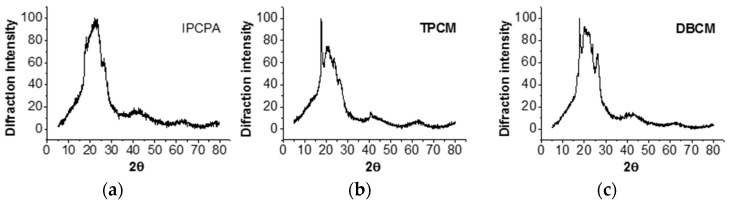
XRD of (**a**) IPCP-A, (**b**) TPCM, and (**c**) DBCM.

The crystallinity of IPCP-A, TPCM, and DBCM films was evaluated (Figure 4), and the X-ray diffraction (XRD) spectra showed an amorphous pattern together with some crystalline regions corresponding to the rigid moieties. These are more evident in TPCM and DBCM polymers, due to the molecular packaging of the *para*-oriented rigid segments.

With these results, the average molecular interchain spacing (<*R*>) can be approximated for amorphous regions, using the equation: <*R*> = 5/8(λ/sin θ*_max_*), where θ*_max_* is the maximum of the X-ray diffraction. The average distance of the polymer chains in the amorphous regions was 4.97, 5.29, and 5.35 Å for IPCP-A, TPCM, and DBCM, respectively. These distances are higher than for commercial poly(*m*-phenylene isophthalamide—4.69 Å) [25], as a result of the introduction of flexible domains, resulting in lower interchain interactions.

**Figure 5:**
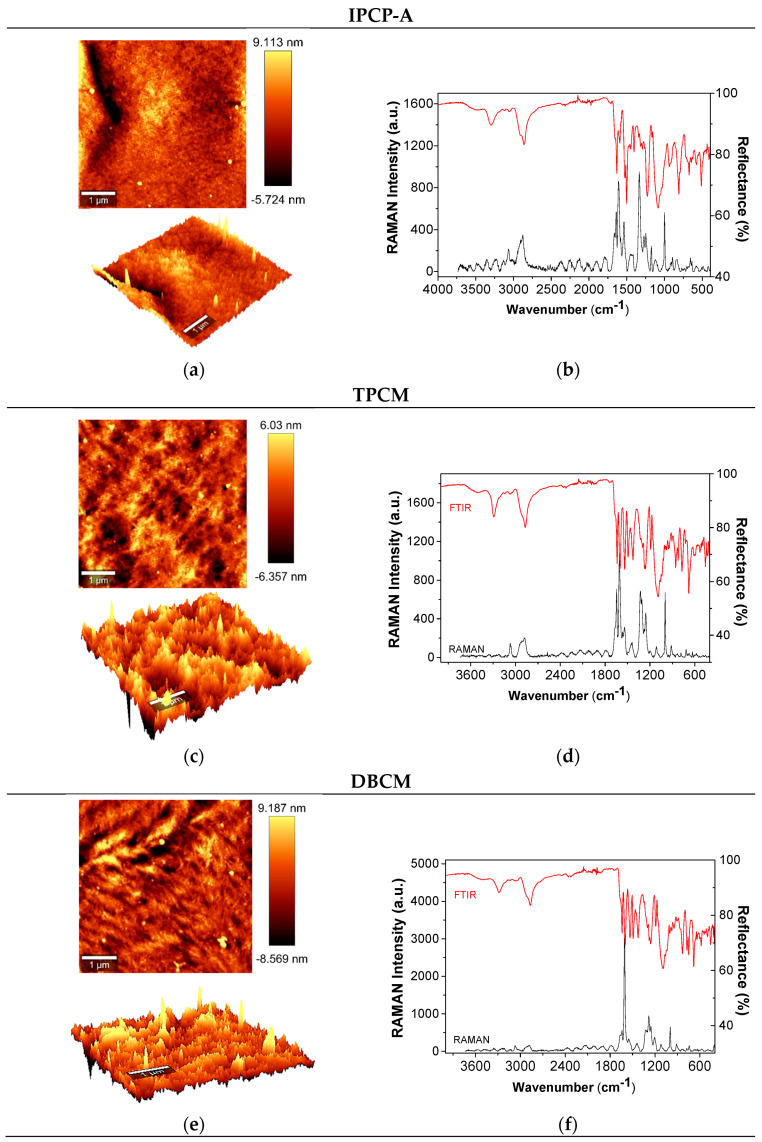
AFM topography images (surface and 3D) in tapping mode of (**a**) IPCP-A, (**c**) TPCM, and (**e**) DBCM. FTIR and RAMAN spectra of (**b**) IPCP-A, (**d**) TPCM, and (**f**) DBCM.

The surface of the films was also characterized with AFM, RAMAN, and FTIR spectroscopy (Figure 5). FTIR and Raman spectra confirm the structure of the polymers. Bands around 3300 and 1650 cm^−1^ corresponding to N–H stretching and the amide carbonyl absorption, respectively, appear in all the spectra of the polymers. Conjugated double bonds of the aromatic rings agree with the substitution of the –NH–Ar–O– aromatic ring around 1600 and 1500 cm^−1^. The strong band around 1050 cm^−1^ is related to the C–O–C vibration of the ether group. Raman bands correspond to prohibited transitions for the infrared spectroscopy, thus showing complementary information.

Topography 3D images in the AFM characterization show the soft blocks (black) and harder blocks (yellow). Soft and hard regions appear to be distributed homogeneously in the surface of the films for all the polymers. The penetration of the AFM tip in the soft regions varied between −8.59 and 9.18 nm, whereas in hard blocks the tip did not penetrate.

**Figure 6:**
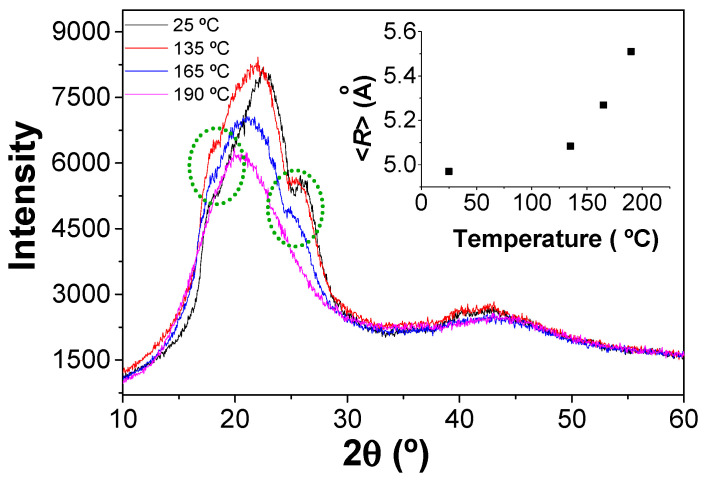
XRD spectra of IPCP-A film at different temperatures. Inset: the relationship between the interchain spacing and the temperature of the DRX.

Additionally, the evolution of the crystalline structure of polyamide films can be characterized by combining the XRD spectra with the AFM surface topography images. In the case of the IPCP-A film, the DSC curve (Figure 3b) shows four different regions delimited from the three thermal transitions detected. The glass transition temperature is around 50 °C, crystallization occurs at 135 °C, and finally, melting about 165 °C. Then, four temperatures were selected to collect the XRD spectra and additionally (before glass transition temperature (rt), crystallization peak (135 °C), melting point (165 °C), and above the melting region (190 °C)). Figure 6 presents the XRD spectra collected at rt, 135 °C, 165 °C, and 190 °C for the IPCP-A film. The XRD spectrum at rt shows two small peaks around 17.5° and 27.5° (green circles, Figure 6), corresponding to the presence of crystallinity in the polyamide. Upon increasing the temperature above the melting point (165 °C), the decrease of these peaks is observed, followed by their complete disappearance at 190 °C. When the four XRD spectra are compared, a displacement of the 2θ angles to lower values with the increasing temperature is observed. The average molecular interchain spacing (<*R*>) can be calculated, increasing as the temperature rises. Moreover, it was observed that the XRD spectrum showed the presence of both crystallinity peaks after cooling the sample at rt, demonstrating the crystallization process of the polyamide during cooling.

**Figure 7:**
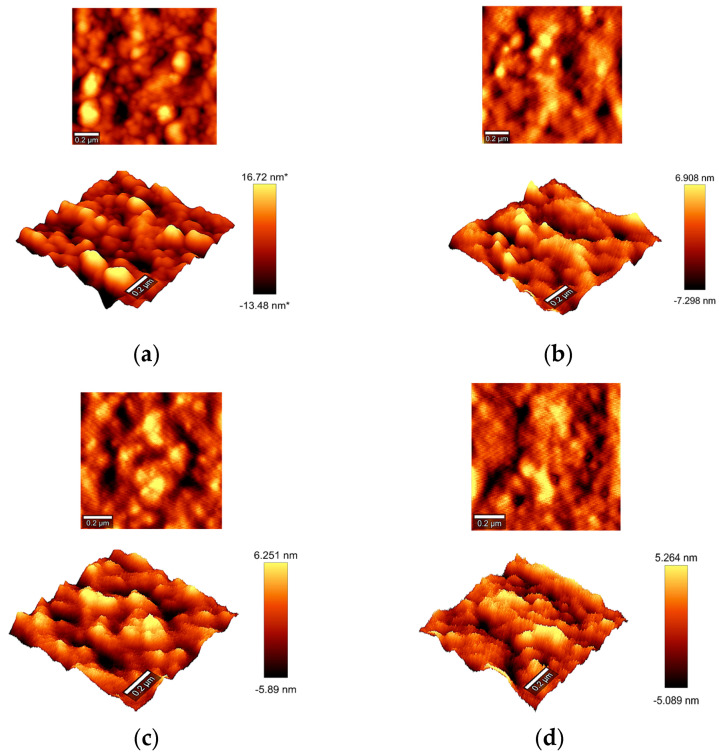
AFM topography images of thermally treated IPCP-A samples (heated and suddenly cooled in salted ice water (−10 °C)): (**a**) nontreated; (**b**) 135 °C; (**c**) 165 °C; (**d**) 190 °C.

In the case of AFM, the technique does not allow for performing the measurements at a given temperature. Accordingly, samples were thermally treated at 135, 165, and 190 °C and then quenched to visualize the influence of the heat treatment on the surface of the IPCP-A (the experimental procedures are described in Section 2). Figure 7 shows the surface topography mapping of each sample after the thermal treatment.

AFM surface pictures show that treating the polyamide at higher temperatures decreases the rugosity of the surface. Differences are more significant when polyamide is treated at 135 °C (crystallization point). At rt, rugosity varies from +17 to −14 nm, and at 135 °C these values are lowered to +7 to −7 nm. When the polyamide is treated at 165 and 190 °C, rugosity does not differ significantly from the one observed at 135 °C (see Figure 7c,d). Concerning the rigid–flexible polymer chain distribution, we did not observe significant differences when samples were treated thermally, and flexible and rigid regions seem to be distributed in channels along the surface. However, it is expected that this distribution could effectively change with temperature. However, these observations were not carried out due to the impossibility of performing AFM measurements at different temperatures in the same sample.

## 4. Conclusions

Polydisperse aromatic diamines containing a dispersion of oxyethylene units in the monomer structure can be prepared in a high yield by an inexpensive general method. The novel diamines can be used as condensation monomers combined with different aromatic diacyl chlorides to give high-molecular-mass poly(ether amide)s, which are, in fact, copolymers consisting of rigid aromatic amide sequences and aliphatic PEO moieties. The relationship between the structure—determined by X-ray, AFM-RAMAN, GPC, and NMR—and the mechanical, thermal, and solubility behavior of the polymers was studied. The polymers show improved flexiblity due to a random distribution of spacers between rigid aromatic amide groups. In addition, they show lower T_g_s, lower crystallinity, and higher solubility than those having a fixed number of PEO sequences in the polymeric structural unit, consequently improving their transformability and expanding their applications. A wide variety of end-properties are possible through the proper combination of the aramid and the polyether segments, enabling the preparation of a la carte materials for many applications.

## Figures and Tables

**Table 1:** Structure of the synthesized polymers.

Polymer	n_4_ + 1	Average Ethyleneoxide Sequences	Ar_1_	Ar_2_
TPCP-A	7.22	8.22	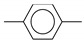	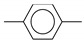
TPCP-B	6.14	7.14		
IPCP-A	7.22	8.22	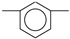	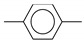
IPCP-B	6.14	7.14		
BDCP-A	7.22	8.22	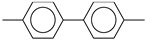	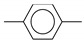
BDCP-B	6.14	7.14		
OBEP-A	7.22	8.22	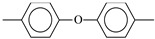	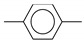
OBEP-B	6.14	7.14		
TPCM	7.22	8.22	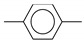	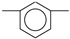
IPCM	7.22	8.22	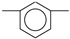	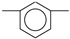
BDCM	7.22	8.22	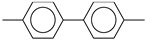	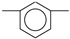
OBEM	7.22	8.22	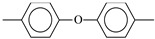	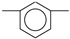

**Table 2:** Viscosities, thermal properties, molecular mass, and polydispersity of poly(ether amide)s.

	Viscosity	Thermal Properties (°C)				Molecular Mass (Dalton)		
Polymer	η_inh_	T_g_	T_m_	T_d_	Residue, % at 650 °C	M_n_	Mw	Mw/M_n_
TPCP-A	0.62	-	259	385	19	7000	15,000	2.1
TPCP-B	0.59	-	277	385	16	7000	16,000	2.3
IPCP-A	0.56	46	161	380	18	7000	13,000	1.9
IPCP-B	0.70	60	191	380	15	7000	15,000	2.2
BDCP-A	1.19	-	297	385	19	14,000	28,000	2.0
BDCP-B	0.94	-	315	385	16	8000	16,000	2.0
OBEP-A	0.49	-	223	385	18	6000	12,000	2.0
OBEP-B	1.21	-	253	385	17	16,000	31,000	1.9
TPCM	0.44	46	157	380	19	15,500	25,500	1.6
IPCM	0.46	44	-	380	20	15,000	24,000	1.6
DBCM	0.49	53	156	385	22	27,000	45,000	1.7
OBEM	0.53	49	-	380	20	16,000	26,000	1.6

**Table 3:** Solubility of poly(ether amide)s (++ = soluble at room temperature; + = soluble on heating; +− = partially soluble; − = insoluble).

Polymer	SOLVENTS					
	Cyclohexanone	Acetone, THF	Acetic Acid	DMF, DMA	NMP	CHCl_3_/TFA
TPCP−A	+	−	−	+	++	++
TPCP−B	+−	−	−	+	+	++
IPCP−A	+	−	+	++	++	++
IPCP−B	+−	−	+−	++	++	++
BDCP−A	+−	−	−	+	+	++
BDCP−B	−	−	−	+−	+	++
OBEP−A	+	−	+	+	++	++
OBEP−B	+−	−	−	+	++	++
TPCM	+	−	+	++	++	++
IPCM	+	+−	+	++	++	++
DBCM	+	−	+−	++	++	++
OBEM	+	+−	+	++	++	++

**Table 4:** Mechanical properties of IPCP-A, DBCM, and TPCM.

Sample	Young’s Modulus (MPa)	Tensile Strength (MPa)	Elongation at Break (%)
IPCP-A	28.3	1.63	24
DBCM	35.6	1.72	31
TPCM	27.1	1.77	37

## Data Availability

The raw/processed data required to reproduce these findings cannot be shared at this time as the data also forms part of an ongoing study.
